# Genome-wide DNA methylation profiling identifies epigenetic signatures of gastric cardiac intestinal metaplasia

**DOI:** 10.1186/s12967-020-02453-2

**Published:** 2020-07-31

**Authors:** Runhua Lin, Chenxi Li, Zhaohui Liu, Ruinuan Wu, Jianghong Lu

**Affiliations:** 1 Department of Pathology, Shantou University Medical College, Shantou, 515041 China; 2 The Second People’s Hospital of Shenzhen/The First Affiliated Hospital of Shenzhen University, Shenzhen, China

**Keywords:** Intestinal metaplasia, Gastric cardia, Genome-wide DNA methylation

## Abstract

**Background:**

Measuring the DNA methylome may offer the opportunity to identify novel disease biomarkers and insights into disease mechanisms. Although aberrant DNA methylation has been investigated in many human cancers and precancerous lesions, the DNA methylation landscape of gastric cardiac intestinal metaplasia (IM) remains unknown. Therefore, we aimed to investigate the genome-wide DNA methylation landscape and to search for potential epigenetic biomarkers of gastric cardiac IM.

**Methods:**

Histopathologic profiling was performed on a total of 118 gastric cardiac biopsies from cancer-free individuals. Genome-wide DNA methylation analysis was performed on 11 gastric cardiac mucosal biopsies (IM = 7; normal = 4) using Illumina 850K microarrays. Transcriptional relevance of any candidate epigenetic biomarker was validated by qRT-PCR.

**Results:**

The detection rate of gastric cardiac IM was 23% (27/118) in cancer-free individuals. Genome-wide DNA methylation profiling showed a global decrease in methylation in IM compared with normal tissues (median methylation = 0.64 and 0.70 for gastric cardiac IM and normal tissues, respectively). Differential methylation analysis between gastric cardiac IM and normal tissues identified 38,237 differentially methylated probes (DMPs) with a majority of sites showing hypermethylation in IM compared with normal tissues (56.3% vs. 43.7%). Subsequent analysis revealed a significant enrichment of hypermethylated DMPs in promoter and CpG islands (*p *< 0.001 for both, Pearson *χ*^2^ test). For DMPs located in promoter CpG islands showing extreme hypermethylation, the candidate gene with the largest number of DMPs (*n* = 7) was mapped to *HOXA5*. Accordingly, mRNA expression of HOXA5 was significantly reduced in IM compared to normal tissue.

**Conclusions:**

Our results suggest the implication of alterations in DNA methylation in gastric cardiac IM and highlight that *HOXA5* hypermethylation may be a promising epigenetic biomarker, emphasizing the role of aberrant HOXA5 expression in the pathogenesis of gastric cardiac IM.

## Background

Intestinal metaplasia (IM) in the gastric mucosa is characterized by emergence of an intestinal-like phenotype (goblet cells and enterocytes). Although there is some controversy about this, the precancerous nature of IM is suggested by the observation that patients affected by IM have a higher risk of developing gastric cancer (GC) than those without IM. There is an annual incidence of GC of 0.25% for IM subjects within 5 years of follow-up [1]. Recently, a meta-analysis study comprising 21 studies has systematically evaluated the risk of GC among individuals with IM and demonstrated a higher risk of GC in IM patients compared with participants without IM (pooled OR = 3.58, 95% CI 2.71–4.73) [2]. Similarly, Barrett’s esophagus (BE), an intestinal metaplasia of the distal esophagus, has been reported to be a premalignant condition conferring an 11.3-fold (95% CI 8.8–14.4) increased risk for esophageal adenocarcinoma (EAC) [3]. Therefore, IM appears to be an important and possibly obligate pathologic stage of tumorigenesis. It would be informative to identify molecular changes responsible for IM development as well as carcinogenesis using IM tissues. Current knowledge of the underlying causes of IM is still incomplete.

DNA methylation—the addition of a methyl group to the CpG dinucleotide, is one of the major epigenetic alterations involving gene expression regulation and chromosomal instability. Aberrant DNA methylation has been demonstrated as a frequent event in a variety of human cancers [4–6], including GC [7, 8]. Moreover, altered DNA methylation is detectable even in gastric IM and dysplasia [9]. Specific DNA methylation changes can serve as detection biomarkers for IM. However, little is known about the comprehensive DNA methylation profile of IMs occurring in the gastric cardia, a special anatomical location within 2 cm below the gastroesophageal junction (GEJ). Most studies to date have investigated epigenetic alterations of individual molecules using tumor-adjacent IM tissues. Nevertheless, this commonly-used approach has limitations in unraveling the methylome of IM in nature. One key factor that likely affects the value of biomarkers identified in tumor-adjacent tissues is the proximity of IM to the tumor. Even non-cancerous gastric epithelial mucosa with normal morphology obtained from GC patients can be considered precancerous because the molecular changes are similar to those associated with GC [10]. Hence, it is important to note that the ideal epigenetic markers of gastric cardiac IM should be identified in IM tissues obtained from patients without cancer prior to any malignant transformation.

In this study, we performed genome-wide DNA methylation analyses in a total of 11 human gastric cardiac mucosa biopsies (normal = 4, IM = 7) from cancer-free individuals using the Human Methylation EPIC platform (Illumina, San Diego, CA) to characterize the DNA methylation signature of gastric cardiac IM. By comparing with normal tissue, we found that the *HOXA5* gene exhibited both promoter hypermethylation and reduced mRNA expression in gastric cardiac IM. Identification of significant methylation events, which occur in gastric cardiac IM preceding the development of malignancy, may provide key molecular insights into the pathogenesis of IM and translate into clinical biomarkers allowing for early detection.

## Methods

### Study design and participants

**Fig. 1:**
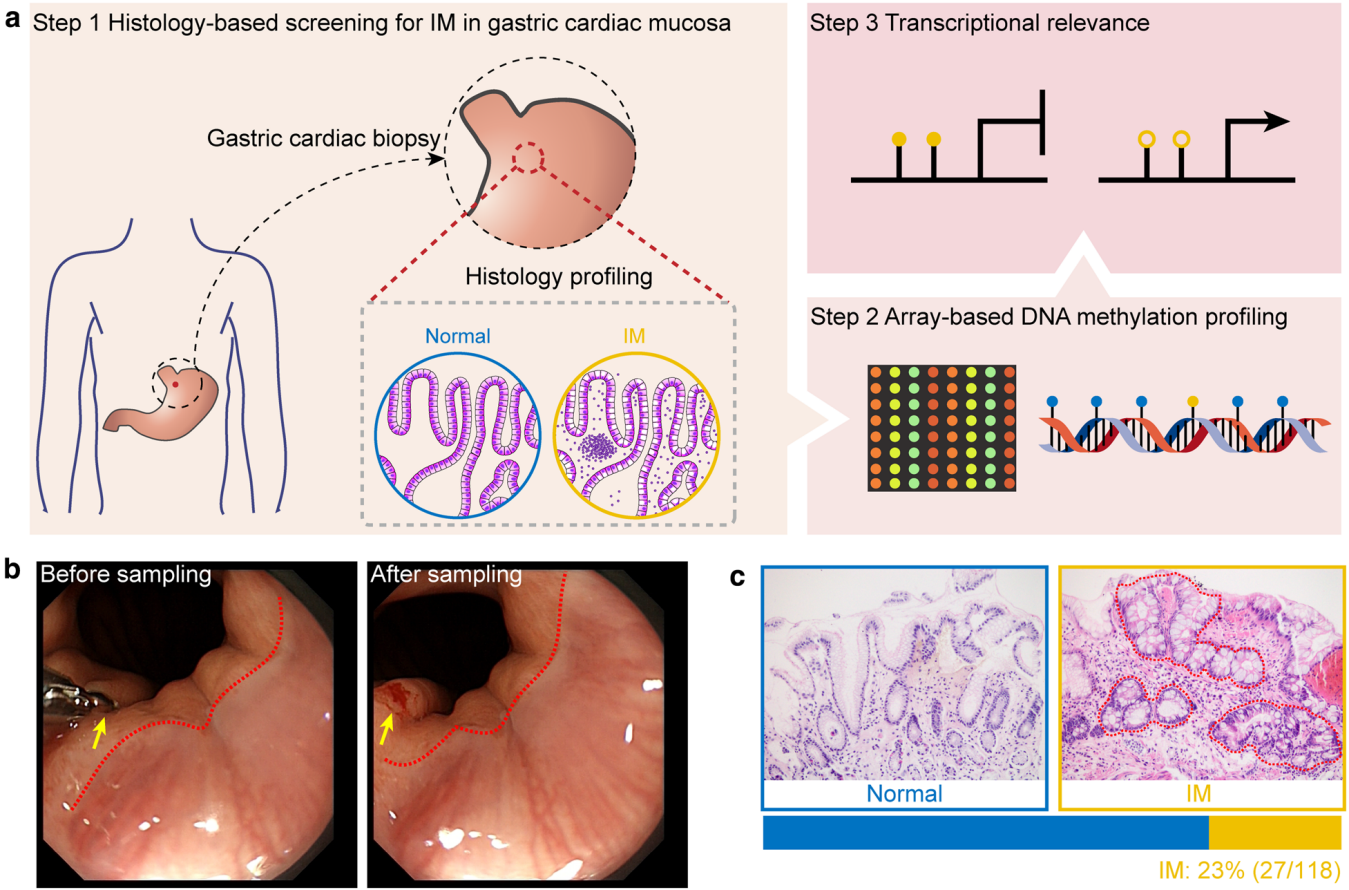
Histology profiling in gastric cardiac mucosa from tumor-free individuals. **a** Flow chart illustrating study design. Step 1, Gastric cardiac mucosa biopsies were obtained from a total of 150 tumor-free individuals. Tissue samples were then stratified into two groups (Normal and IM) based on histopathology. Red dashed circle denotes the biopsy location. Step 2, A total of 11 freshly frozen samples (four normal controls and seven IMs) were assayed for DNA methylation profiling to identify IM-specific DNA methylation markers. Step 3, Verification of IM-specific DNA methylation markers at the transcriptional level. Yellow closed and open circles indicate methylated and unmethylated CpGs, respectively. **b** Representative endoscopic images illustrating the biopsy location of the gastric cardiac mucosa (indicated by yellow arrows). Before, endoscopic image showing gastric cardiac mucosa before sampling. After, endoscopic image showing gastric cardiac mucosa after sampling. Red dashed lines denote the gastro-esophageal junction. **c** Hematoxylin and eosin-stained sections show the normal (upper left) and IM (upper right) gastric cardiac mucosa. The intestinal metaplastic epithelium is composed of goblet cells interspersed between mucous cells, both at the surface and in the glandular epithelium (outlined by red dashed lines). Horizontal slice bar depicts the proportion of histology-based detected IM in gastric cardia in a total of 118 tissue samples. Source data are presented in Additional file 2: Table S1

A total of 118 cancer-free participants were recruited at The Second People’s Hospital of Shenzhen in China during the period from March 2019 to May 2019. Gastric cardiac mucosal biopsies were obtained by a single experienced gastroenterologist. For each individual, at least two gastric cardiac biopsies were obtained. One biopsy was fixed in buffered formalin and embedded in paraffin for histopathology; the other one was immediately frozen and stored at − 80 °C until being thawed for DNA and RNA extraction. A flow chart demonstrating study design is depicted in Fig. 1a. All endoscopic specimens were divided into two categories based on histopathology: normal gastric cardiac mucosa (Normal) and intestinal metaplasia (IM). The presence of goblet cells, absorptive (brush border) cells and/or Paneth cells was used to define IM. For genome-wide DNA methylation profiling, 11 (IM = 7; normal = 4) of the 118 gastric cardiac mucosal samples were processed for EPIC DNA methylation microarray analysis. For expression analysis of any candidate gene, 22 (IM = 11; normal = 11) available RNA samples of the 118 cancer-free subjects were processed for qRT-PCR analysis.

This study was approved by the institutional review board of the participating institutions. Informed consent was obtained from all participants.

### Sample selection procedure for DNA methylation and RNA transcription analyses

All 118 endoscopic specimens were divided into two groups based on morphology: normal and intestinal metaplasia. These samples showed slight differences even within the same group. For example, there were different degrees of inflammatory infiltration present in tissues in the normal group. Likewise, there were different degrees of intestinal metaplasia among samples in the IM group. For mRNA expression analysis, we focused on relatively homogeneous morphology within the same group. To do this, we selected samples showing normal glandular structure (percentage of glandular cells > 80%) without evident inflammatory infiltration serve as the normal group (11 out of 91), and samples representing IM (11 out of 27) should have abundant intestinal metaplastic lesions (i.e., percentage of metaplastic glands > 80% by histology). For genome-wide DNA methylation analysis, we also took other factors into account, such as gender and age. We finally selected a total of 11 samples with available RNA (7 out of 11 IM samples and 4 out of 11 normal samples) for methylome analysis (Additional file 1: Figure S1).

### DNA and RNA extraction

Genomic DNA and total RNA from fresh-frozen samples were isolated using the AllPrep DNA/RNA Mini Kit (Qiagen, Germany). All procedures were conducted according to the manufacturer’s protocol. Histopathology was confirmed by H&E for each fresh-frozen sample.

### Illumina 850K (EPIC) DNA methylation array

Bisulfite-converted genomic DNA was used to hybridize the Illumina’s Infinium MethylationEPIC BeadChips (Illumina, San Diego, CA, USA). Illumina iScan was used to scan the BeadChips. The raw intensity data (IDAT) were imported into R (3.6.0; https://cran.r-project.org/) and then analyzed with the Chip Analysis Methylation Pipeline (*ChAMP*) package (2.14.0) [11] for data preprocessing, normalization, and comparison between groups. Singular value decomposition analysis was performed to identify confounding factors. Briefly, raw data were preprocessed using the *minfi* package (1.30.0) [12] and normalized for technical variations using SWAN [13]. We removed poorly performing probes with a detection *p* > 0.01 in one or more samples (*n* = 2622), probes with less than three beads, and probes with a missing value in more than one sample (*n* = 14,889). Cross-reactive probes were removed (*n* = 11), as well as probes with single nucleotide polymorphisms (SNPs) [14] (*n* = 95,965). Finally, we removed probes on X or Y chromosomes (*n* = 16,470) to avoid any gender-specific methylation bias. This resulted in a final dataset of 733,015 autosomal probes (866,895 in total) for downstream analyses.

### Differential methylation analysis

Methylation at each CpG was represented as “*β* values” (ranging from 0 to 1), where 0.0 is equivalent to 0% methylation and 1.0 is equivalent to 100% methylation at a given CpG dinucleotide [15]. Statistical analyses were performed on *β* values. Mean *β* values were calculated for cases (IM) and control (Normal). The mean Δ*β* was calculated by subtracting the mean *β* value of controls from that of cases. Accordingly, a positive Δ*β* value denoted relative hypermethylation, and a negative Δ*β* value indicated hypomethylation in IMs. Probe-wise differential methylation analysis was carried out using the *limma* (3.40.2) package with sex and age as covariates [16]. Differentially methylated probes (DMPs) were identified by comparing mean *β* values in the IM group to the mean *β* values in the Normal group for a particular CpG site. We set the criteria for DMPs as calling significance of a Benjamini–Hochberg adjusted *p* < 0.01 and a difference in *β* value between groups larger than 0.2 (i.e., |Δ*β*| > 0.2). This threshold yielded a final list of 38,237 DMPs between IM and normal subjects. All processing of array data and analyses were conducted in RStudio (Version 1.2.1335) using an R environment (version 3.6.0).

### Distribution analysis DMPs

The DMPs were classified into different groups in terms of their distributions relative to gene regions (promoter, gene body, 3′UTR, or intergenic region) and CpG island regions (island, shore, shelf, or open sea) according to 850K array annotation. The expected counts were calculated with the 733,015 probes remaining after filtering. Statistics were calculated using a multinomial goodness-of-fit Chi squared test. As post hoc tests to evaluate which category drives an effect, additional Chi squared tests were run on each category versus the sum of all the other categories.

### Selection of candidate differentially methylated genes (DMGs)

Despite the considerable variability in methylation associated with gastric IM, we screened our data to identify potential function-related epigenetic biomarkers of IM. We applied filtering criteria to our list of 38,237 DMPs found in the CpG site-level group differences test to select candidate CpGs with large and replicable differences in methylation levels. We selected DMGs based on the following step-wise criteria: (1) DMPs that were annotated in both promoter regions (TSS1500, TSS200, 5′UTR, and first exon) and CpG islands (CGIs); (2) DMPs with a mean methylation level in IM tissue greater than 0.7 (extremely high methylation) or less than 0.3 (extremely low methylation): theoretically, extremely high or low DNA methylation levels are more likely to be associated with loss of expression or abundant expression, respectively, so this strategy will facilitate the identification of possible biomarkers with functional relevance in disease phenotype; and (3) genes with multiple (i.e., at least three) DMPs. After filtering, we selected 13 DMPs mapping to three genes that matched these criteria.

### Reverse-transcription PCR and quantitative real-time PCR

For reverse transcription, cDNA was synthesized from 500 ng of RNA (*n* = 10) using the PrimeScript RT reagent kit (Takara, Tokyo, Japan) according to the manufacturers’ instructions. Quantitative PCR was carried out using cDNA dilutions ranging between neat to 1:100. All reactions were performed using ABI 7500 PRISM™ SDS (Applied Biosystems, Foster City, CA) with RQ software. Primer sequences are available upon request. All reactions were run in triplicate, and the threshold numbers were averaged. The absence of contamination was verified by “no template” controls. For expression analysis in IM and normal gastric cardiac tissue, HOXA5 expression was normalized to the mean Ct values of normal samples, and mRNA levels in the IM samples were displayed as a ratio relative to the normal levels. The fold change was calculated using the 2^−ΔΔCt^ method with *β*-actin as an endogenous control. The expression level was considered to be downregulated if the mRNA expression fold was ≤ 0.5 in comparison with the normal tissue.

### Statistical analysis

Statistical analyses of the data were performed in RStudio (Version 1.2.1335) (http://www.rstudio.com/) using an R environment (version 3.6.0) (https://www.R-project.org). Infinium probes showing significant differences in DNA methylation levels between seven IM and four normal gastric cardiac samples in the discovery cohort were identified using a limma package. A false discovery rate (FDR) < 0.01 was considered significant. Unsupervised hierarchical clustering (Euclidean distance, Ward method) based on DNA methylation levels of the 11 samples in the cohort was performed.

## Results

### Detection rate of gastric cardiac IM in cancer-free individuals

According to the overall study design (Fig. 1a), histologic studies were conducted in a total of 118 gastric cardiac mucosal biopsies from cancer-free individuals. The corresponding baseline data of the study participants are summarized in Additional file 2: Table S1. As shown in Fig. 1b, all gastric cardiac mucosal biopsies showed normal gastroscopy. However, histology-proven IM was presented in 23% (27/118) of the individuals (Fig. 1c), suggesting that IM is a common pathological change in the gastric cardia.

### Global DNA methylation profile of gastric cardiac IM

**Fig. 2:**
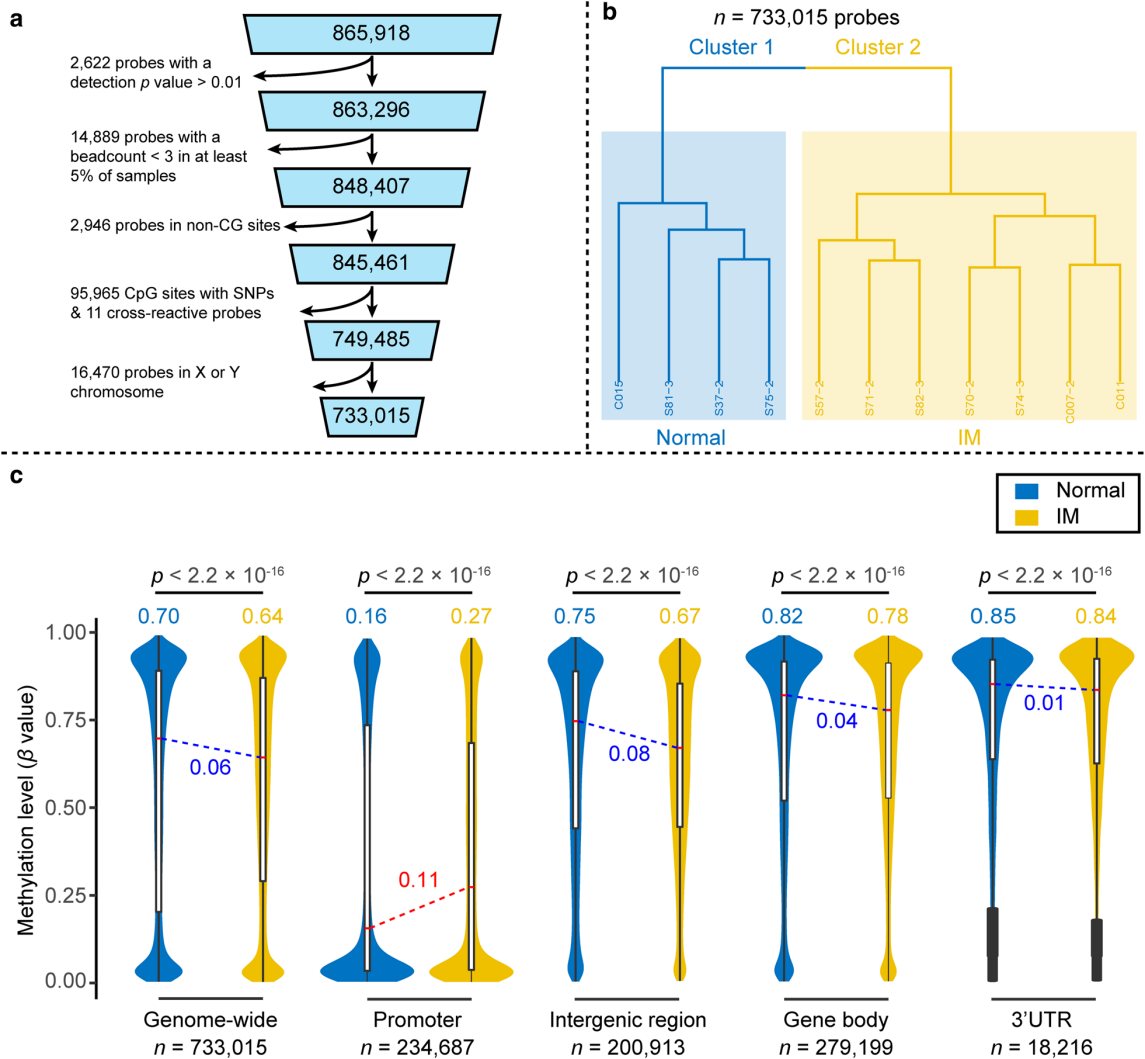
Global DNA methylation profiling of gastric cardiac IM. **a** Flow chart for filtering pipeline used to generate a set of high-confidence probes. A total of 733,015 finalized probes were generated. **b** Clustering dendrogram from unsupervised hierarchical clustering based on overall methylation status. This analysis separates samples into two clusters discriminated also by histology classification (Normal and IM). **c** DNA methylation levels for different genomic locations. Violin plots show DNA methylation on genome-scale (*n* = 733,015), gene promoters (*n* = 234,687), intergenic regions (*n* = 200,913), gene body regions (*n* = 279,199), and 3′UTRs (*n* = 18,216). Box plots within each violin plot indicate the interquartile range, and the red horizontal lines denote the median methylation. In all cases, the *y*-axis represents the methylation level on a 0 to 1 scale (i.e., 0 to 100%)

To unravel the genome-wide DNA methylation landscape of gastric cardiac IM, we performed DNA methylation analysis on DNA samples from normal gastric cardiac mucosa (normal = 4) and intestinal metaplastic mucosa (IM = 7) using Illumina Human Methylation EPIC microarrays. Based on the selection procedure, we finally selected a total of 11 samples from the 118 specimens (IM = 7; Normal = 4) for methylome analysis (Additional file 1: Figure S1). General demographic data of the 11 individuals are summarized in Additional file 2: Table S1. Following quality control and preprocessing (see the Materials and Methods section), a set of 733,015 probes remained for further analyses (Fig. 2a). To get an overview of methylation patterns in the data, we applied unsupervised hierarchical clustering to the overall probes (*n* = 733,015) for the whole genome of all samples, both IM and normal tissues. This analysis separated samples into two clusters that discriminated also by histology classification (i.e., clear distinction between IM and normal samples; Fig. 2b).

The overall DNA methylation patterns followed a bimodal distribution, with high (> 80%) or low (< 20%) levels of DNA methylation in the majority of CpG sites in both IMs and normal controls. Separating CpGs in gene context based on genomic features displayed a distinct pattern: the majority of unmethylated (< 20%) CpGs were restricted to gene promoters (Fig. 2c). The distribution of DNA methylation in specific gene regions was similar in both groups. Global genome-wide methylation analysis demonstrated that the global methylation level in IMs was lower than that in normal gastric cardiac tissues (median methylation = 0.64 and 0.70, respectively, *p* < 2.2 × 10^−16^, Wilcoxon rank test; Fig. 2c). However, when the CpG sites were analyzed separately based on gene regions, we found significantly different methylation levels between normal and IM tissues. Indeed, hypomethylation of IMs was particularly prominent in intergenic regions, with an 8% median methylation reduction in IMs. Strikingly, DNA methylation in gene promoters demonstrated an increased 9% median methylation in IMs compared with normal controls. These results indicate significant DNA methylation alterations in IM development.

### Hypermethylated DMPs are highly enriched in gene promoters and CpG islands

**Fig. 3:**
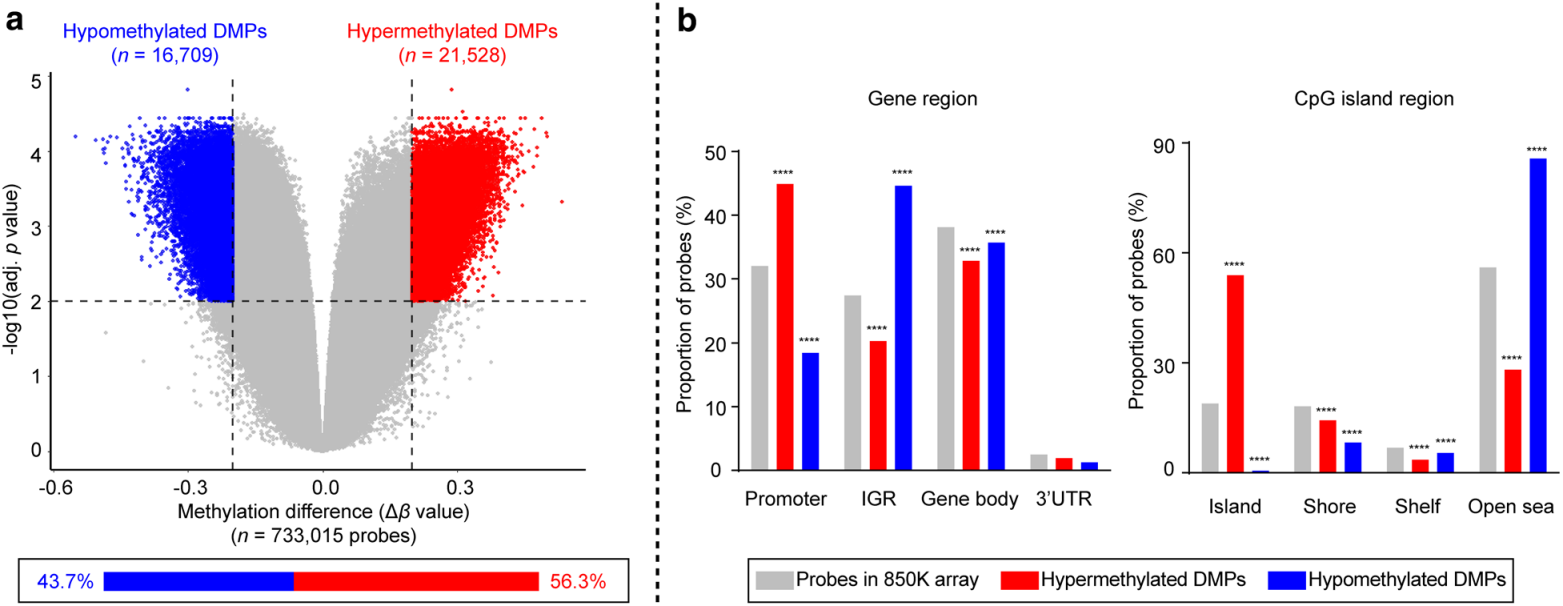
Differentially methylated probes in gastric cardiac IM. **a** Volcano plot of probe-level methylation in IM versus normal. The plot shows the relationship between magnitude of difference in *β* values (Δ*β* values; *x*-axis) and adjusted *p* values (negative log_10_ transformed adjusted *p* values; *y*-axis). Each dot represents a single probe. The cutoff of adjusted *p* = 0.01 and 20% in methylation difference (|Δ*β*| = 0.2) are marked by horizontal and vertical dashed lines, respectively. Red and blue dots denote hyper- and hypomethylated DMPs, respectively. The percentages of hyper- and hypomethylated DMPs are displayed (bottom). Source data are provided in Additional file 3: Table S2. **b** Bar charts showing the distribution of DMPs in relation to gene region (left panel) and CpG island region (right panel). The distribution of CpG probes was as follows: the total probes in the 850K array available for analysis (grey; *n* = 733,015 probes), hypermethylated DMPs (red; *n* = 21,528 probes), and hypomethylated DMPs (blue; *n* = 16,709 probes) according to gene region (left panel) and CpG island region (right panel). *P* values were calculated by Chi squared tests (****p < 0.0001). Source data are provided in Additional file 4: Table S3

We next set out to identify DNA methylation alterations specific to IM. By comparing gastric cardiac IM to normal tissue (IM vs. normal), we found a total of 38,237 differentially methylated probes (DMPs; adjusted *p* value < 0.01, |Δβ| > 0.2; Additional file 3: Table S2). These represented 20.9% of the total CpG probes (*n* = 733,015). Most of the DMPs (56.3%) were hypermethylated in IMs (21,528 hyper- and 16,709 hypomethylated; Fig. 3a).

Given that genomic location plays an important role in sculpting DNA methylation landscape and mediating its effects, we next sought to ascertain the genomic distribution of DMPs. Overall, DMPs showed a significantly different distribution than the proportions presented in the 850K array (hypermethylated DMPs: *χ*^2^ = 17,382, *p* < 2.2 × 10^−16^; hypomethylated DMPs: *χ*^2^ = 6571.1, *p* < 2.2 × 10^−16^). Furthermore, we found a striking enrichment of hypermethylated DMPs in gene promoters (44.9%) and significant overrepresentation of hypomethylated DMPs located in intergenic regions (44.6%) (*p *< 0.0001 for both, Pearson *χ*^2^ test; Fig. 3b left panel). With respect to CpG island regions (island, shore, shelf, and open sea), hypermethylated DMPs were significantly overrepresented in CpG islands (53.9%) (*p *< 0.0001, Pearson *χ*^*2*^ test), whereas hypomethylated DMPs were enriched in open sea regions (85.7%) and underrepresented in CpG islands (0.5%) (*p *< 0.0001 for both, Pearson *χ*^2^ test, Fig. 3b right panel, Additional file 4: Table S3). These findings suggest the important role of aberrant hypermethylation of promoter CpG islands in gastric cardiac IM development.

### Hypermethylation of *HOXA5* is a candidate biomarker of gastric cardiac IM

We have established a substantial predominance of hypermethylated DMPs in promoter CpG islands. To identify biologically meaningful biomarkers, we next focused exclusively on DMPs located in promoter CpG islands (i.e., DMPs in TSS1500, TSS200, 5′UTR, or 1st Exon regions). As a result, a total of 6784 DMPs mapping to 1883 genes (differentially methylated genes, DMGs) were identified (Additional file 5: Table S4).

**Fig. 4:**
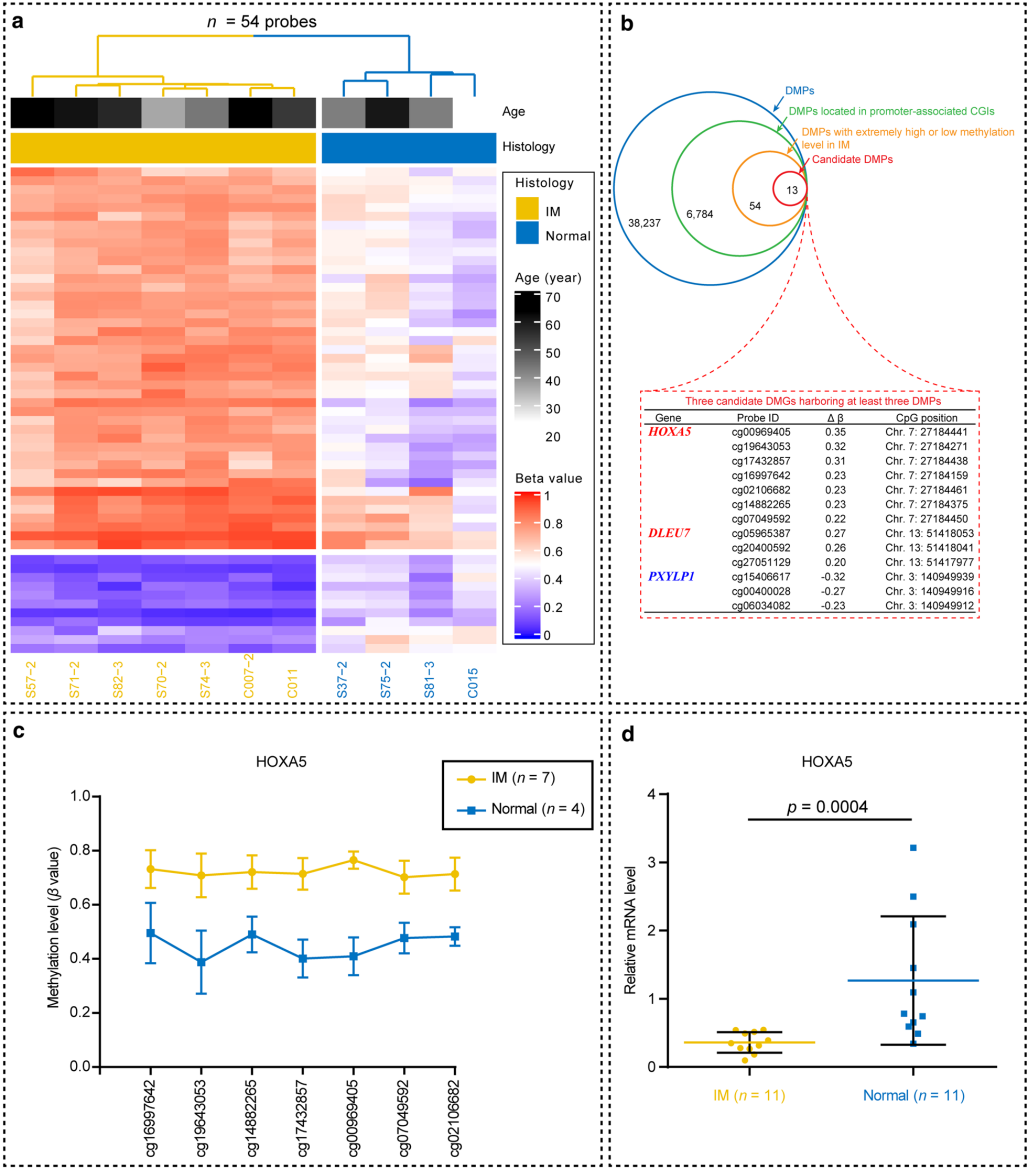
Identification of candidate differentially methylated genes in gastric cardiac IM. **a** Unsupervised hierarchical cluster heatmap of selected DMPs in promoter CpG islands. A total of 54 DMPs showed extremely high (mean *β* > 0.7 in IM and mean *β* < 0.7 in normal) or extremely low (mean *β* < 0.3 in IM and mean *β* > 0.3 in normal) levels of DNA methylation. Of these, 43 CpG sites (79.6%) were hypermethylated, and 11 (20.4%) were hypomethylated. Note that IM and normal control are distinctly divided by unsupervised hierarchical clustering. Each row represents a CpG probe, and each column indicates an independent sample. The cells are colored according to methylation level (blue = unmethylated, red = fully methylated) at each CpG in each sample, as described by the color key. For each sample (column), the methylation levels are plotted (bottom), and the corresponding histology and age is shown in the bars directly above the heatmap. **b** Diagram showing the number of DMPs identified in each step by using the extra data filtering strategy. Three genes, *HOXA5*, *DLEU7*, and *PXYLP1*, harboring multiple DMPs (i.e., ≥ 3 DMPs identified in a single gene) were selected for validation and are indicated in the red dotted rectangle. **c** Differentially methylated levels in neighboring CpGs of *HOXA5*. Each data point represents the mean *β* value of the group, and error bars show SDs. The *y*-axis shows the absolute methylated fraction (*β* value) of each CpG site; the *x*-axis shows the CpG ID coordinates. Error bars represent means and standard deviations. **d** Relative mRNA expression of IM (*n* = 11) and normal (*n* = 11) gastric cardiac tissue (*n* = 11) measured by quantitative real-time PCR. Gene expression in IM was normalized against that in normal tissue. The mean value (± SD) is shown. *P* value was calculated by Mann–Whitney *U* test. Error bars represent standard deviations

To narrow down the list of DMGs, we filtered our data based on the methylation level for each of the DMPs (DMPs with a mean *β* > 0.7 in IM and a mean *β* < 0.7 in Normal for hypermethylated DMGs; DMPs with a mean *β* < 0.3 in IM and a mean *β* > 0.3 in Normal for hypomethylated DMGs). This strategy resulted in 54 CpG sites (43 hypermethylated DMPs and 11 hypomethylated DMPs) corresponding to 33 unique candidate hypermethylated DMGs and 8 hypomethylated DMGs (Fig. 4a). We noted three genes harboring multiple DMPs (≥ 3 DMPs), out of which two genes showed hypermethylation (*HOXA5* [7 CpGs] and *DLEU7* [3 CpGs]), and one showed hypomethylation (*PXYLP1* [3 CpGs]) (Fig. 4b). Of note, *HOXA5* exhibited the largest number of DMPs in IM tissue. It had seven CpG sites (cg16997642, cg19643053, cg14882265, cg17432857, cg00969405, cg07049592, and cg02106682), which pointed to the TSS1500 region (Fig. 4c). Hypermethylation of multiple consecutive CpGs in promoter CpG islands has been reported as a critical mechanism by which genes may be silenced [17]. Additionally, we identified hypermethylation of the *HOXA5* gene as a promising biomarker of intestinal metaplasia biomarker for the following reasons: (1) HOXA5 acts as tumor-suppressing role during the development and progression of gastric cancer [18]; (2) Loss of HOXA5 function induces a developmental defect causing Clara cells to transdifferentiated into goblet cells in the lung epithelia [19]. Hence, we examined the mRNA expression level of *HOXA5* in 22 cancer-free individuals (IM = 11, normal = 11, Additional file 1: Figure S1). Consistent with methylation data, qRT-PCR analysis revealed a significant down-regulated expression of HOXA5 in IM compared with normal tissue (Fig. 4d).

## Discussion

To our knowledge, this is the first comprehensive study providing distinct DNA methylation profiles of naturally occurring gastric cardiac IM in samples from subjects without cancer, based on genome-wide analysis. We observed significant changes in DNA methylation and identified hypermethylation in the promotor of *HOXA5* as epigenetic biomarker of gastric cardiac IM. Our results suggest the importance of DNA methylation modification to the development of gastric cardiac IM.

The current results highlight the relevance of DNA methylation alterations in the natural history of gastric cardiac IM. For each sample, DNA was obtained from gastric cardiac mucosal biopsies without malignant lesions. The use of a cancer-free cohort, representing only histologically proven IM, offered a unique opportunity to investigate DNA methylation status in IM prior to malignancy. We showed that gastric cardiac IM and normal gastric cardiac tissues exhibited distinct DNA methylation profiles; the methylation profiles of IM and normal tissues could be clearly distinguished from one another. There is ample evidence regarding cancer-specific DNA methylation patterns [20–23]. Moreover, several reports [24–26] have revealed that significant DNA methylation alterations occur in precancerous lesions. These facts support the notion that changes in DNA methylation could be promising biomarkers for early disease detection. However, we still have little information concerning comprehensive gastric cardiac IM-specific DNA methylation. In this context, our results revealed that DNA methylation was largely increased in promoter regions. However, we have identified decreased methylation levels in intergenic regions as well as genome-wide hypomethylation. This observation is similar to that seen in BE [27] and cancer [28] methylomes. Regardless of a functional effect or not, our data suggest that aberrant DNA methylation occurs early in IM tissue and is potentially applicable in clinical practice.

At the DMP level, we found common IM-specific methylation patterns consisting of 38,237 CpG sites (DMPs) that were significantly different from those of the normal tissues. Most of the DMPs in IM were hypermethylated (21,528, 56.3%) rather than hypomethylated (16,709, 43.7%). In addition, DMPs in the promoter regions (TSS1500, TSS200, 5′UTR, and 1st exon regions) and CGIs were more likely to be hypermethylated than outside these regions. By contrast, the hypomethylated DMPs preferentially localized in intergenic regions and open seas (Fig. 3b). These data are consistent with the findings from a published array-based BE methylation study [29], suggesting that DNA hypermethylation in promoter-associated CGIs is a common feature of metaplastic processes and that at least some of the high-confidence hypermethylated DMPs in these regions probably have a pathogenic role in IM formation.

The use of a new, high-content methylation array allowed us to identify a substantially larger number of DMPs than was previously possible. Because no universally accepted strategy exists for analyzing Infinium methylation arrays, we used extra data-filtering steps (Fig. 4b) in searching DMGs due to the large number of DMPs. By focusing on genes with multiple DMPs located in promoter CpG islands, we finally identified two extremely hypermethylated (*β* value > 0.7 in IM) DMGs (*HOXA5* and *DLEU7*), and one hypomethylated (*β* value < 0.3 in IM) DMG (*PXYLP1*). HOXA5 is a transcriptional factor that plays key roles in regulating human embryonic development and adult stem cell differentiation [30], and a loss of HOXA5 function can perturb intestinal maturation in mice [31]. It has been revealed that HOXA5 protein represses the aggressiveness of colon cancer [32] and breast cancer [33]. These studies have suggested that *HOXA5* may be a broad-spectrum tumor suppressor gene with the potential for wide-ranging clinical significance and applications. Hypermethylation of *HOXA5* has been reported in several cancer types [34, 35], and in these cancers it shows reduced expression [18]. Recently, *HOXA5* was found to be the most differentially hypermethylated gene between gastric cancer and tumor-adjacent gastric tissue [36]. The present study is the first to our knowledge to report aberrant hypermethylation of *HOXA5* in gastric cardiac IM. The effect of hypermethylation on the *HOXA5* promoter seems to be functional, for quantitative PCR analysis reveals a decrease in *HOXA5* expression in IM compared with normal tissues. This finding warrants further functional studies to elucidate whether methylation-induced silencing of *HOXA5* is a driver event for gastric cardiac IM development.

There are two main limitations that need to be noted in this study. First, the small sample size and the lack of an independent validation set, meaning that our results must be interpreted with caution. Nevertheless, the candidate DMGs identified in our results (e.g., the hypermethylation of promoter CpG islands in IM) are consistent with known biology. These candidates were confirmed to have down-regulated mRNA by qRT-PCR, indicating that the candidate DMGs found in this study are not artifacts. Second, although biopsies were collected from cancer-free individuals to model the cascade from normal to IM, longitudinal studies based on monitoring a patient during the development from normal to an intestinal metaplastic lesion are necessary to confirm and clarify the nature of gastric cardiac IM.

## Conclusions

In summary, this comprehensive study shows that, even in the context of a small sample size, DNA methylation profiling can separate the gastric cardiac IM tissues from normal ones. Our data highlight *HOXA5*, which, in addition to promoter hypermethylation, also shows significantly reduced mRNA expression, suggesting that aberrant hypermethylation of *HOXA5* might have a role in gastric cardiac IM development. This needs to be addressed in further functional studies. The robust data obtained from this study are valuable in improving our understanding of aberrant DNA methylation in the pathogenesis of IM.

## Supplementary information

**Additional file 1: Figure S1.** Sample selection procedure in pathologic assessment, mRNA expression analysis, and DNA methylation profiling.

**Additional file 2: Table S1.** General characteristics of the study subjects.

**Additional file 3: Table S2.** List of IM-related differentially methylated CpGs comparing gastric cardiac IM and normal tissue.

**Additional file 4: Table S3.** Genomic distribution of the probes in different groups.

**Additional file 5: Table S4.** List of 6784 DMPs located in promoter-associated CpG islands.

## Data Availability

The data support the findings of this study are available from the corresponding author upon reasonable request.

## Abbreviations

IM: Intestinal metaplasia
BE: Barrett’s esophagus
GEJ: Gastroesophageal junction
GC: Gastric cancer
DMPs: Differentially methylated probes
UTR: Untranslated region
PCR: Polymerase chain reaction
