# Supplementary figures and images for: Genome-wide DNA methylation profiling identifies epigenetic signatures of gastric cardiac intestinal metaplasia
Source: J Transl Med. 2020 Jul 31;18:292. doi: 10.1186/s12967-020-02453-2 (PMC7393819; doi:10.1186/s12967-020-02453-2)

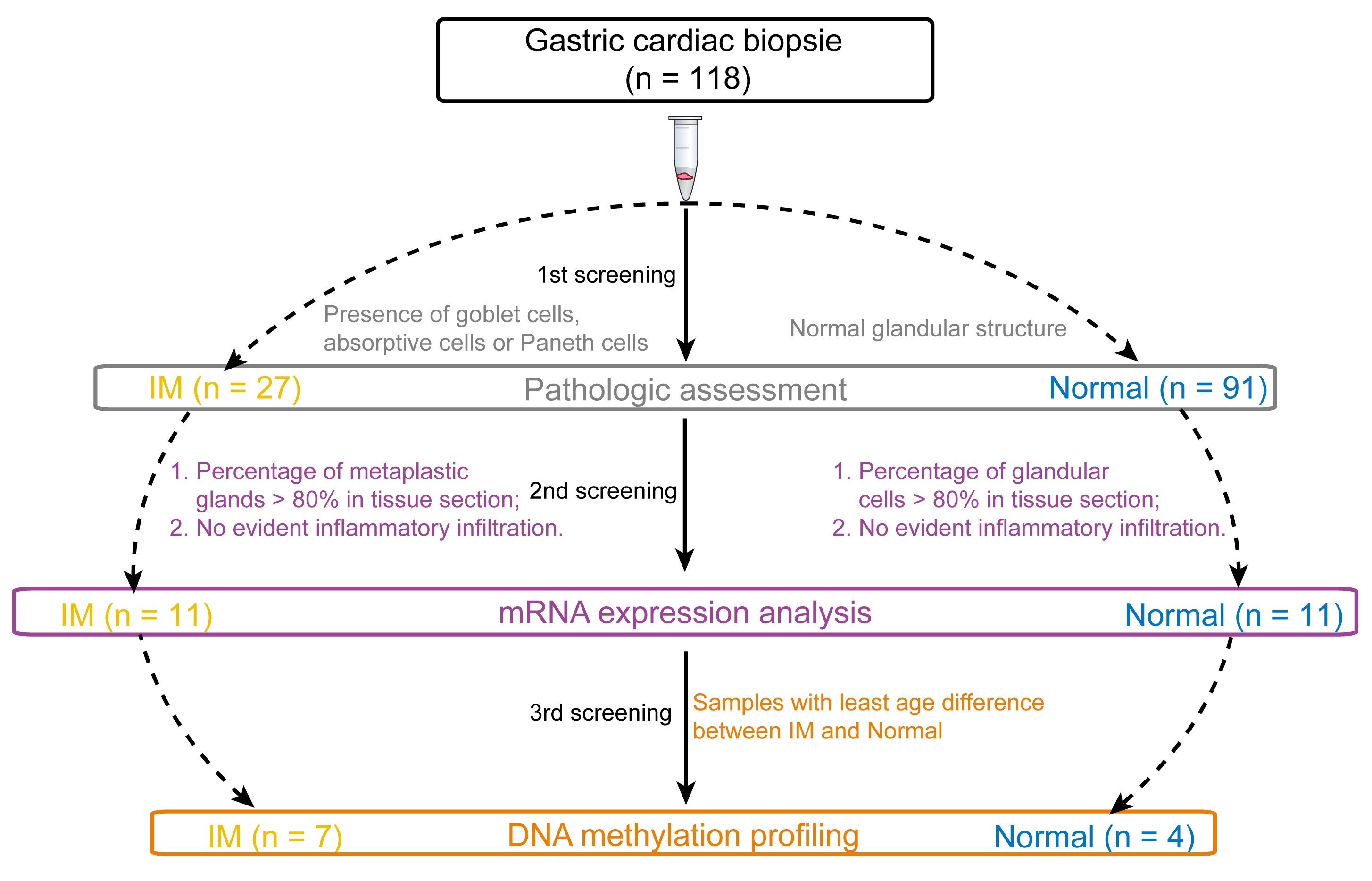

Supplement: Supplementary file 1 — Additional file 1: Figure S1. Sample selection procedure in pathologic assessment, mRNA expression analysis, and DNA methylation profiling. [file 12967_2020_2453_MOESM1_ESM.tif]
